# Chemotherapy improves distant control in localized high-grade soft tissue sarcoma of the extremity/trunk

**DOI:** 10.1186/s13569-020-00132-w

**Published:** 2020-07-09

**Authors:** Victoria T. Rizk, Arash O. Naghavi, Andrew S. Brohl, David M. Joyce, Odion Binitie, Youngchul Kim, John P. Hanna, Jennifer Swank, Ricardo J. Gonzalez, Damon R. Reed, Mihaela Druta

**Affiliations:** 1 Department of Hematology and Oncology, Moffitt Cancer Center and Research Institute, 12902 Magnolia Drive, Tampa, FL 33612 USA; 2 Department of Radiation Oncology, Moffitt Cancer Center and Research Institute, Tampa, FL USA; 3 Department of Sarcoma, Moffitt Cancer Center and Research Institute, Tampa, FL USA; 4 Department of Biostatistics and Bioinformatics, Moffitt Cancer Center and Research Institute, Tampa, FL USA; 5 Department of Surgery, University of South Florida, Tampa, FL USA; 6 Department of Pharmacy, Moffitt Cancer Center and Research Institute, Tampa, FL USA

**Keywords:** Soft tissue sarcoma, Localized, Chemotherapy, Distant control

## Abstract

**Background:**

Soft tissue sarcomas (STS) are rare and heterogeneous tumors making chemotherapy use controversial. Our goal was to identify a subset of patients with primary STS that benefit with the addition of chemotherapy.

**Methods:**

A retrospective chart review included intermediate to high-grade localized primary STS of the extremity/trunk, and tumor size > 5 cm. The effect of chemotherapy was evaluated for local control (LC), distant control (DC), progression free survival (PFS), and overall survival (OS).

**Results:**

In this cohort (n = 273), patients were treated with surgery (98%), radiation (81%), and chemotherapy (24.5%). With a median follow-up of 51 months, the entire cohort’s 5-year LC, DC, PFS, and OS are 79.1%, 59.9%, 43.8%, and 68.7%, respectively. The addition of chemotherapy did not provide a DC benefit (p = 0.238) for the entire cohort. High-grade disease (n = 210) experienced a 5-year benefit in DC (68% vs. 54.4%, p = 0.04), which was more pronounced with MAI (Mesna, Adriamycin, Ifosfamide) based regimens (74.2%, p = 0.016), and a 5-year PFS (50.8% vs 45%, p = 0.025) and OS benefit (76.2% vs 70%, p = 0.067) vs. no chemotherapy. On multivariate analysis of the high-grade subset, chemotherapy independently predicted for a DC benefit (HR 0.48 95% CI 0.26–89, p = 0.019). The benefit of chemotherapy was more pronounced with MAI, showing a significant benefit in DC (HR 0.333 95% CI 0.145–0.767, p = 0.01) and PFS (HR 0.52 95% CI 0.28–0.99, p = 0.047).

**Conclusion:**

In patients with localized STS > 5 cm, the high-grade subset had a distant control benefit with the addition of chemotherapy, leading to improved progression free survival. This is more pronounced with the use of MAI and should be considered in patients eligible for this regimen.

## Background

Sarcomas are a heterogeneous group of mesenchymal neoplasms that account for 1% of all adult malignancies with more than 50 histologic subtypes identified and a reported incidence of 13,040 cases in 2018 [1, 2]. Approximately 60% of soft tissue sarcomas (STS) are diagnosed in the extremity or trunk [3]. The primary treatment modalities include surgery, radiation, and chemotherapy. Despite this trimodal approach, patients frequently experience local and distant recurrence. The risk of distant recurrence has been associated with various tumor characteristics, including: high-grade, size > 5 cm, anatomic site, histology, depth, and local recurrence [4, 5].

Although low histologic grade and tumors measuring < 5 cm in size may be adequately treated with surgery alone, disease with unfavorable features are generally considered for adjuvant treatment [6, 7]. Due to the heterogeneity and rarity of the disease, clinical studies evaluating the role of chemotherapy for patients at a high-risk of distant metastasis (e.g., high grade, > 5 cm, and often tumor depth) have led to inconsistent results, and its use is therefore not well defined in current guidelines [8, 9].

Given the ongoing controversy regarding the role of systemic therapy for high-risk localized soft tissue sarcoma, we performed a retrospective review of the experience of our large, high-volume sarcoma referral center. The goal of this study was to review and contrast the treatment response of patients seen at the Moffitt Cancer Center (MCC) with intermediate to high-grade, tumor size ≥ 5 cm, STS of the extremities and trunk that received surgery and/or radiation with the response of those that received surgery and/or radiation combined with chemotherapy.

## Methods

A retrospective chart review was performed evaluating patients with STS of the extremity and trunk treated at the MCC from 1998 to 2016. Patients included were ≥ 18 years of age and diagnosed with localized extremity or trunk STS that was ≥ 5 cm size based on pathologic size and intermediate to high-grade on histology (as defined by the Federation Nationale des Centres de Lutte Contre le Cancer or FNCLCC). Patients excluded held a diagnosis of bone or cartilage sarcomas (i.e., osteosarcoma, Ewing Sarcoma, etc.), rhabdomyosarcoma, site of disease located on head/neck or abdomen/pelvis, metastatic disease at time of diagnosis, and well-differentiated on pathology (e.g., FNCLCC Grade 1). This data was collected from the Cancer Registry Source System, a large database at Moffitt Cancer Center. Of the 522 patients in the original dataset, 273 patients met inclusion criteria.

Patient, tumor, and treatment characteristics evaluated included: gender, primary site(s) of disease, tumor size, performance status, histology, and initial treatment (i.e., chemotherapy, radiation, or surgery). In the chemotherapy cohort, variables such as agents, dosages, and number of cycles were recorded. Grade 3 toxicity data (as defined by Common Terminology Criteria for Adverse Events v5.0) were collected, including neurotoxicity, GI toxicity (i.e., nausea/vomiting), infectious disease complications (i.e., neutropenic fever), renal impairment, myelosuppression, and cardiotoxicity. Any reasons for dose reduction and/or discontinuation of chemotherapy were also recorded. Subset analysis was performed on clinical features that pose an even higher risk of distant metastasis (size ≥ 8 cm and/or FNCLCC Grade 3), to identify those who would most benefit from chemotherapy.

## Statistical methods

Patient and tumor characteristics were compared in the chemotherapy and non-chemotherapy cohorts with the use of Pearson Chi square, Fisher’s Exact Test, and Mann-U-Whitney as appropriate.

Time-to-event outcomes are defined from the date of diagnosis to the date of event, with censorship at last follow-up. These variables include time to local control (LC), distant control (DC), any recurrence or death (progression free survival or PFS), and overall survival (OS). Recurrence was an event in LC, DC, and PFS, whereas death was an event in PFS and OS. Patients that progressed distantly during neoadjuvant treatment and did not undergo surgery were considered an event. Univariate analyses were performed using a cox-regression analysis, with trending and significant variables defined as a 2-sided *p* value of 0.1 and 0.05, respectively. Time-to-event outcomes were illustrated with Kaplan–Meier based curves and comparisons were made with the use of log-rank, univariate analyses was also performed with cox regression analysis.

Multivariate cox regression analysis was performed including age (> 70 vs. ≤ 70), tumor size (≥ 8 cm vs. < 8 cm), ECOG performance status (2+ vs. 0–1), sex, chemotherapy, histology (reference: sarcoma not otherwise specified), and disease site. The multivariate analysis was performed for the entire cohort, grade 3 subset, and grade 3 patients treated with MAI/MAID (vs. no chemotherapy). The Cox regression analysis was performed for local control, distant control, progression free survival, and overall survival. As MAI based chemotherapy regimens are becoming standard practice in STS [6, 10–12], the use of this chemotherapy regimen in particular was compared to patients that received no chemotherapy. Statistical analysis was performed with SPSS (version 22; IBM Corporation, Armonk, NY).

This review was approved by the MCC and the University of South Florida Institutional Review Boards.

## Results

### Patient characteristics

Of the 522 patients identified with localized, extremity or truncal sarcoma, 273 patients met inclusion criteria. For the entire cohort, there was a median age of 64 years old (range 24–97), follow-up of 51 months (range 3–181), and median tumor size of 11.1 cm (range 5–43.5 cm). The distribution of histologic grading was grade 2 in 63 cases (23%) and grade 3 in 210 (77%). In total, 41 (15%) local and 93 (35%) distant recurrences developed as first events. The cohort primarily consisted of Eastern Cooperative Oncology Group (ECOG) performance status of 0–1 (n = 236, 86.4%), grade 3 (n = 210, 77%), ≥ 8 cm in size (n = 179, 66%), underwent surgery (n = 267, 98%), received radiation (n = 220, 81%), and received chemotherapy (n = 67, 25%) (Table 1). Of the six patients who did not undergo surgery, three (3/6) developed distant progression while receiving neoadjuvant chemotherapy thereby becoming unresectable, and three (3/6) elected to receive radiation over surgery due to unrelated comorbidities.

**Table 1:** Patient and Treatment characteristics of complete cohort

	Total (n = 273)
	No. (%)
Sex	
Female	110 (40.3%)
Male	163 (59.7%)
Histology	
Undifferentiated pleomorphic sarcoma	56 (8.1%)
Undifferentiated spindle cell sarcoma	30 (11%)
Undifferentiated sarcoma nos	22 (20.5%)
Fibrosarcoma	5 (1.8%)
Myxofibrosarcoma	44 (16.1%)
Liposarcoma nos	7 (2.6%)
Myxoid liposarcoma	26 (9.5%)
Pleomorphic liposarcoma	8 (2.9%)
Mixed liposarcoma	8 (2.9%)
Dedifferentiated liposarcoma	16 (5.9%)
Leiomyosarcoma	17 (6.2%)
Myxoid leiomyosarcoma	2 (0.7%)
Synovial sarcoma	23 (8.5%)
Angiosarcoma	5 (1.8%)
Malignant peripheral nerve sheath tumor	3 (1.1%)
Small round cell sarcoma	1 (0.4%)
Surgery	267 (97.8%)
Radiation	220 (80.6%)
Chemotherapy role	
None	206 (75.5%)
Neoadjuvant	56 (20.5%)
Adjuvant	8 (2.9%)
Both	3 (1.1%)
Type of chemotherapy	
None	206 (75.5%)
MAI	53 (19.4%)
MAI → gemcitabine/docetaxel	1 (0.4%)
MAID	5 (1.8%)
Dacarbazine/doxorubicin	4 (1.5%)
Paclitaxel	2 (0.7%)
Gemcitabine/docetaxel	1 (0.4%)
Unknown	1 (0.4%)

	Median (range)
Age at diagnosis (years)	64 (24–97)
Path tumor size (cm)	9.7 (5–43.5)
Median follow-up in living patients (months)	51 (3–181)

Patients receiving chemotherapy tended to be younger (54 years vs. 65 years, p < 0.001), but were otherwise balanced, when compared to the non-chemotherapy arm. The majority of patients receiving chemotherapy were treated with Doxorubicin 60–75 mg/m^2^ and Ifosfamide 8–10 g/m^2^ (n = 53/67) ranging from 1 to 6 cycles (median 2 cycles). Less commonly used regimens were: Doxorubicin, Ifosfamide, and Dacarbazine (750 mg/m^2^) (i.e., MAID) (n = 5/71), as well as Dacarbazine (750–1000 mg/m^2^) and Doxorubicin (60–75 mg/m^2^) (n = 4/71). Single agent paclitaxel (80 mg/m^2^) (n = 2/71) and Gemcitabine (900 mg/m^2^), Docetaxel (100 mg/m^2^) (n = 2/71) were rarely used.

### Institutional outcomes

#### Disease control and survival on univariate analysis

After a median follow-up of 51 months, the 5-year LC, DC, PFS, and OS are 79.1%, 59.9%, 43.8%, and 68.7%, respectively, for the entire cohort.

Factors predictive of a detriment in LC on univariate analysis include older age at diagnosis (HR 1.03 95% CI 1.01–1.05, p = 0.021), poor performance status (ECOG ≥ 2) (HR 2.21 95% CI 1.01–4.81, p = 0.046), and upper extremity tumors (HR 2.092 95% CI 1.08–4.05, p = 0.028). Factors associated with a detriment in DC on univariate analysis include male gender (HR 1.59 95% CI 1.03–2.46, p = 0.037), poor performance status (HR 3.44 95% CI 2.18–5.42, p ≤ 0.001), and larger tumor size (HR 1.04 95% CI 1.01–1.07, p = 0.012). Factors associated with a detriment in PFS include male sex (HR 1.45 95% CI 1.02–2.06, p = 0.036), high-grade (HR 1.79 95% CI 1.13–2.81, p = 0.012), age of diagnosis ≥ 70 (HR 1.54 95% CI 1.10–2.15, p = 0.012), poor performance status (HR 3.51 95% CI 2.38–5.17, p ≤ 0.001), and larger tumor size (HR 1.03 95% CI 1–1.06, p = 0.023). Factors associated with a detriment in OS include high-grade (HR 3.11 95% CI 1.43–6.76, p = 0.004), performance status (HR 3.28 95% CI 2.01–5.32, p < 0.001), age ≥ 70 (HR 1.61 95% CI 1.03–2.5, p = 0.035), and histology (p = 0.002) (Table 2).

**Table 2:** Univariate tumor outcome of complete cohort

	No chemo (n = 206)	Chemo (n = 67)	Univariate analysis p-value
	No. (%)	No. (%)	Chi Square association test
Primary site			
Lower extremity	136 (66%)	50 (75%)	0.224*
Upper extremity	42 (20%)	9 (13%)	
Thorax	14 (7%)	6 (9%)	
Trunk	13 (6%)	1 (2%)	
Overlapping	1 (1%)	1 (2%)	
Tumor size (≥ 8 cm)			
< 8 cm	76 (37%)	18 (27%)	0.133
≥ 8 cm	130 (63%)	49 (73%)	
Sex			
Female	82 (40%)	28 (42%)	0.774
Male	124 (60%)	39 (58%)	
Grade (FNCLCC)			
2	46 (22%)	17 (25%)	0.608
3	160 (78%)	50 (75%)	
Radiation	170 (83%)	50 (75%)	0.156
Surgery	203 (99%)	64 (96%)	0.143
Age			
≥T70	90 (43.7%)	4 (6%)	< 0.001*
< 70	116 (56.3%)	63 (94%)	
ECOG performance status			
0	56 (27%)	14 (21%)	0.644*
1	117 (57%)	49 (73%)	
2	31 (15%)	4 (6%)	
3	2 (1%)	0 (0%)	

*Fisher’s Exact Test

**Fig. 1:**
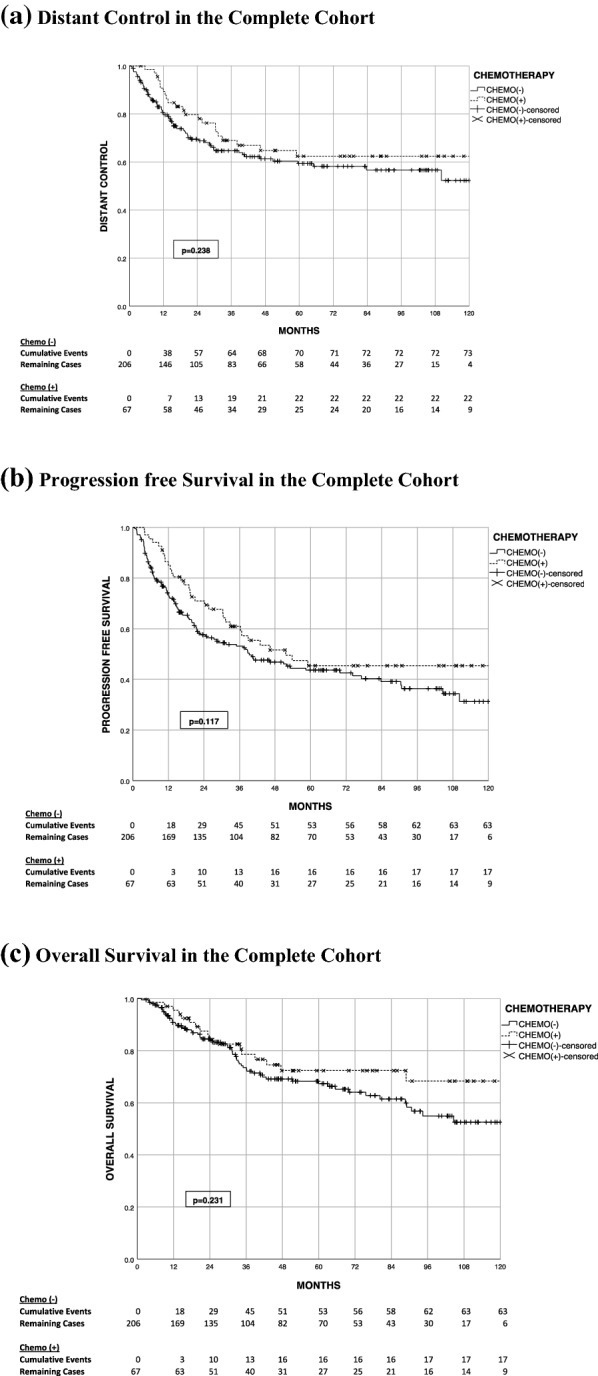
Time-to-event outcomes in the complete cohort. *a* Distant control in the complete cohort. *b* Progression free survival in the complete cohort. *c* Overall survival in the complete cohort

On univariate analysis of the entire cohort, chemotherapy did not benefit LC (p = 0.419), DC (p = 0.238), PFS (p = 0.117), or OS (p = 0.231) (Fig. 1).

#### Independent predictors of outcome (multivariate analysis)

On multivariate analysis, no factors were independently associated with LC. Factors associated with a DC detriment include performance status (ECOG 2+ vs. 0–1; HR 3.521 95% CI 2.046–6.06, p < 0.001) and histology (p = 0.014) (Table 3).

**Table 3:** Multivariate analysis of complete cohort

		ALL		G3		G3 + MAI/MAID	
		p-value	HR (95% CI)	p-value	HR (95% CI)	p-value	HR (95% CI)
Distant control							
Chemotherapy	Chemo(+) (vs. chemo(–))	0.368	0.77 (0.435–1.361)	*0.037*	0.475 (0.236–0.954)	*0.01*	0.333 (0.145–0.767)
Sex	Male (vs. female)	0.172	1.405 (0.863–2.289)	0.142	1.501 (0.873–2.579)	0.229	1.387 (0.814–2.362)
ECOG performance status	2+ (vs. 0–1)	*< 0.001*	3.521 (2.046–6.06)	*< 0.001*	3.654 (2.019–6.612)	*< .001*	3.146 (1.684–5.876)
Age (categorical)	<70 (vs. ≥ 70 yoa)	0.819	1.063 (0.629–1.797)	0.083	1.659 (0.936–2.941)	0.096	1.634 (0.917–2.912)
Pathologic tumor size	≥ 8 cm (vs. < 8 cm)	0.356	1.275 (0.761–2.136)	0.215	1.448 (0.807–2.598)	0.214	1.449 (0.807–2.603)
Histology*		*0.014*	.	0.138	.	*0.035*	.
Primary site*		0.759	.	0.962	.	0.98	.
Progression free survival							
Chemotherapy	Chemo(+) (vs. chemo(–))	0.581	0.876 (0.547–1.402)	0.101	0.629 (0.361–1.095)	*0.047*	0.52 (0.28–0.99)
Sex	Male (vs. female)	0.149	1.326 (0.904–1.945)	0.21	1.306 (0.86–1.981)	0.201	1.32 (0.86–2.01)
ECOG performance status	2 + (vs. 0–1)	*< 0.001*	3.32 (2.123–5.191)	*< 0.001*	2.75 (1.659–4.557)	*< 0.001*	2.83 (1.68–4.77)
Age (categorical)	< 70 (vs. ≥ 70 yoa)	0.315	0.802 (0.521–1.234)	0.625	1.125 (0.702–1.802)	0.527	1.17 (0.72–1.89)
Pathologic tumor size	≥ 8 cm (vs. < 8 cm)	0.788	1.058 (0.703–1.592)	0.592	1.135 (0.715–1.8)	0.533	1.16 (0.72–1.87)
Histology*		*0.019*	.	0.078	.	0.098	.
Primary site*		0.536	.	0.736	.	0.639	.
Overall survival							
Chemotherapy	Chemo(+) (vs. chemo(–))	0.759	0.909 (0.493–1.675)	0.481	0.772 (0.375–1.587)	0.171	0.564 (0.248–1.28)
Sex	Male (vs. female)	0.266	1.337 (0.801–2.233)	0.247	1.389 (0.796–2.423)	0.181	1.464 (0.837–2.558)
ECOG performance status	2+ (vs. 0–1)	*0.001*	2.523 (1.455–4.376)	*0.003*	2.46 (1.364–4.434)	*0.015*	2.179 (1.16–4.094)
Age (categorical)	< 70 (vs. ≥ 70 yoa)	0.277	0.728 (0.411–1.29)	0.356	0.752 (0.41–1.378)	0.616	0.853 (0.459–1.586)
Pathologic tumor size	≥ 8 cm (vs. < 8 cm)	0.947	0.982 (0.567–1.701)	0.631	0.866 (0.483–1.555)	0.835	0.938 (0.513–1.714)
Histology*		*0.037*	.	0.292	.	0.103	.
Primary site*		0.731	.	0.824	.	0.595	.

* p-value designates the entire variables association with outcome on MVA

Histologies that predicted for a detriment in DC include undifferentiated sarcoma (HR 3.047 95% CI 1.056–8.786, p = 0.039) and synovial sarcoma (HR 5.074 95% CI 1.456–17.684, p = 0.011).

Factors that were associated with a detriment to PFS and OS include performance status (RFS: HR 3.32 95% CI 2.123–5.191, p < 0.001; OS: HR 2.523 95% CI 1.455–4.376, p = 0.001) and histology (p = 0.019; p = 0.037).

Histologies that predicted for a detriment in PFS include undifferentiated sarcoma (HR 3.049 95% CI 1.231–7.552, p = 0.016), spindle cell sarcoma (HR 2.713 95% CI 1.075–6.847, p = 0.035), leiomyosarcoma (HR 3.005 95% CI 1.036–8.719, p = 0.043), synovial sarcoma (HR 3.732 95% CI 1.196–11.648, p = 0.023), angiosarcoma (HR 5.163 95% CI 1.268–21.02, p = 0.022), and malignant peripheral nerve sheath tumor (HR 7.852 95% CI 1.877–32.85, p = 0.005).

Histologies that predicted for a detriment in OS include fibrous histiocytoma (HR 3.969 95% CI 1.079–14.602, p = 0.038) and malignant peripheral nerve sheath tumor (HR 8.619 95% CI 1.572–47.271, p = 0.013).

#### Chemotherapy toxicity

In the cohort of patients receiving chemotherapy, the majority (64%) of patients receiving chemotherapy did not experience any clinically significant grade 3 adverse events (Table 4). The most common grade 3+ toxicities observed included: myelosuppression (24%), GI toxicity (10%), neurotoxicity (5%), and cardiotoxicity (2%).

**Table 4:** Chemotherapy toxicity

Grade 3 adverse events	No. (%)
None	43 (64%)
Ifosfamide neurotoxicity	3 (4%)
Nausea/vomiting	2 (3%)
Anemia requiring epo	3 (4%)
Neutropenic fever	13 (19%)
Diverticulitis	2 (3%)
Transaminitis	0 (0%)
Cardiotoxicity	1 (2%)
Mucositis	3 (3%)

### Subset analysis

#### Identifying subsets associated with a distant control benefit

**Fig. 2:**
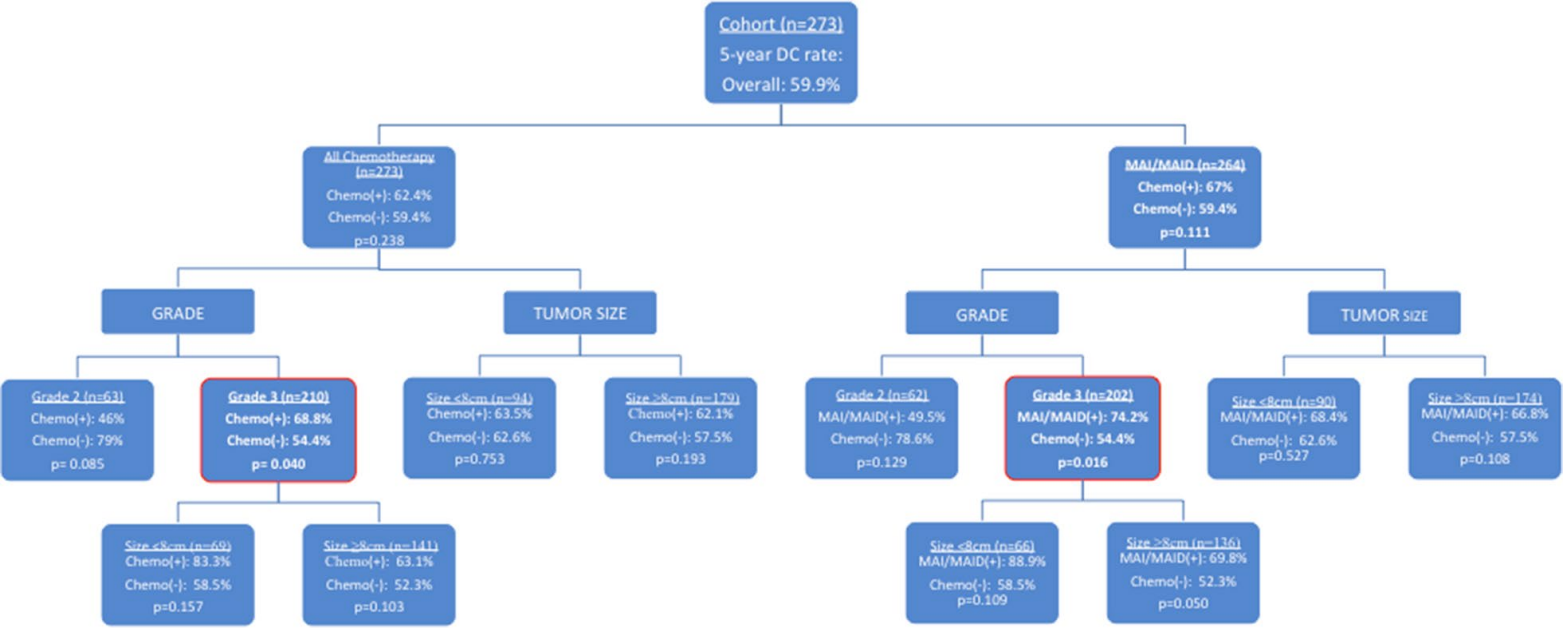
Summary of 5 year distant control in subsets evaluated

**Fig. 3:**
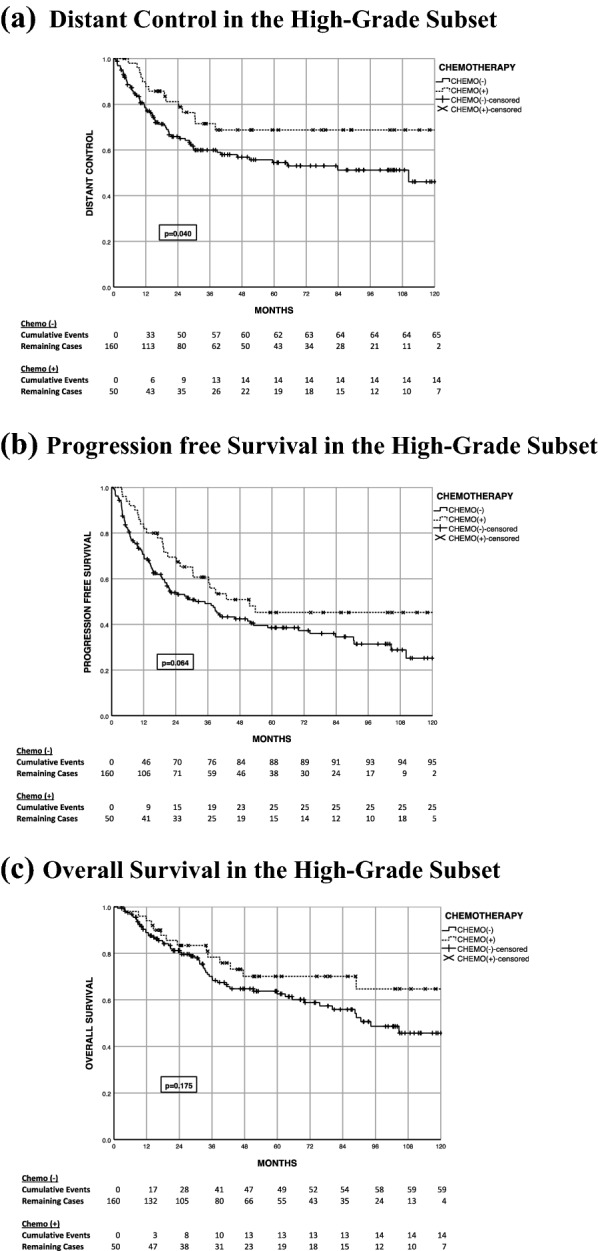
Time-to-event outcomes in the high-grade subset. **a** Distant control in the high-grade subset. **b** Progression free survival in the high-grade subset. **c** Overall survival in the high-grade subset

Kaplan–Meier curves evaluating subsets based on FNCLCC grade (2 vs. 3), tumor size (< 8 cm vs. ≥ 8 cm), and chemotherapy were used (all vs. MAI based) (Fig. 1). In high-grade (FNCLCC grade 3) disease, there was a significant improvement in distant control for those patients who received chemotherapy (Fig. 2). Although there is no significant distant control benefit with the use of chemotherapy for the entire cohort (p = 0.238), subset analysis showed that MAI/MAID chemotherapy had a non-significant improvement in the 5-year DC (67% vs. 59.4%, p = 0.111, n = 264). Specifically in patients with high-grade disease (n = 202), the use of MAI/MAID chemotherapy provided a significant 5-year DC benefit (74.2% vs. 54.4%, p = 0.016, n = 202), when compared to the non-chemotherapy group (Fig. 3).

#### Univariate predictors of outcome in high-grade and large tumor subsets

Subset analysis evaluating cohorts with high-grade disease (n = 210) or tumor ≥ 8 cm (n = 179) in size were performed. On univariate analysis of the high-grade cohort, chemotherapy predicted for a significant DC benefit (HR 0.55 95% CI 0.31–0.98, p = 0.043), but the impact on PFS (HR 0.66 95% CI 0.43–1.03, p = 0.066) and OS (HR 0.68 95% CI 0.38–1.19, p = 0.178) did not reach statistical significance. Cohorts stratified by size alone did not identify a subset where chemotherapy significantly improved distant control.

**Fig. 4:**
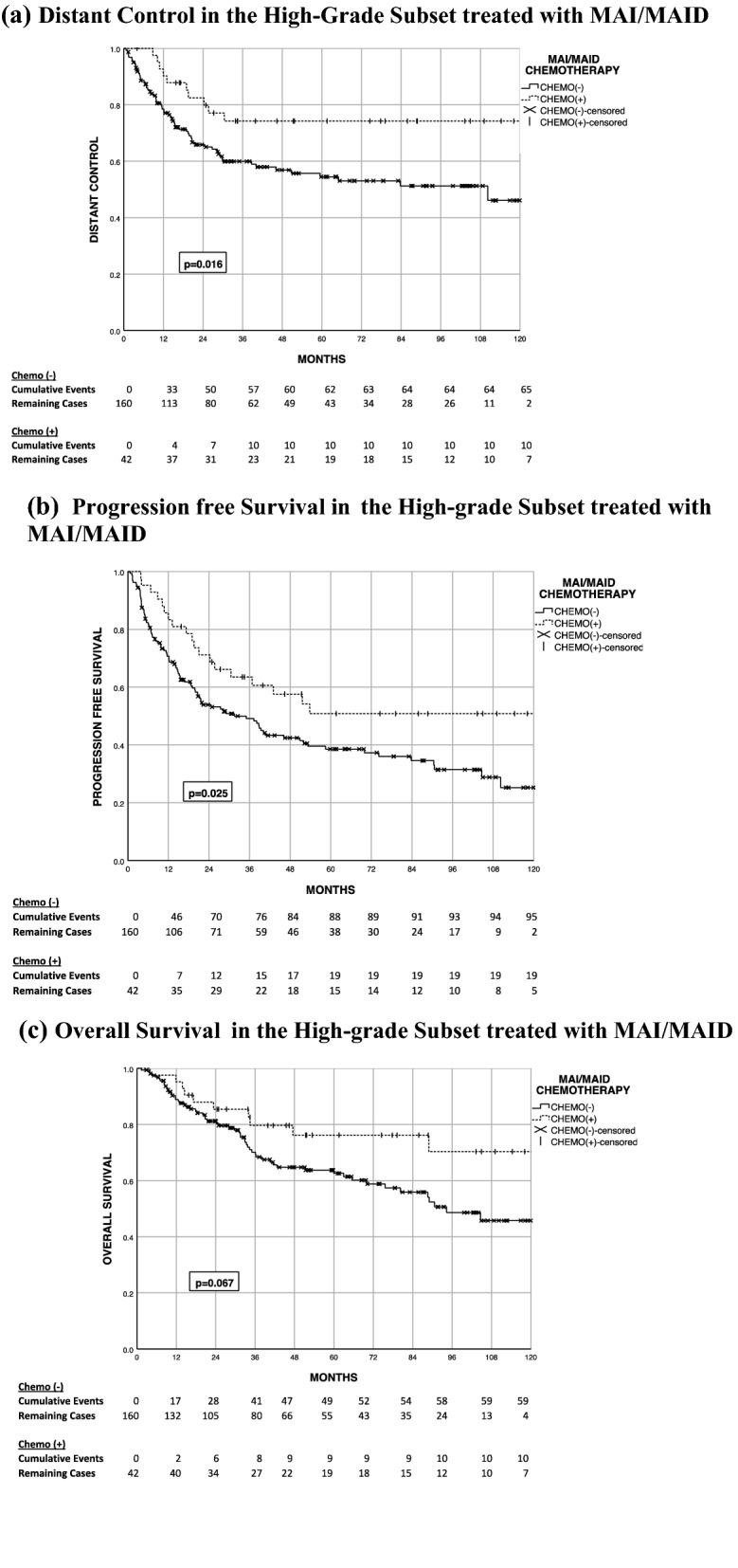
Time-to-event outcomes in the high-grade subset treated with MAI/MAID. **a** Distant control in the high-grade subset treated with MAI/MAID. **b** Progression free survival in the high-grade subset treated with MAI/MAID. **c** Overall survival in the high-grade subset treated with MAI/MAID

Further subset analysis was performed on the chemotherapy cohort to evaluate outcome differences for high-grade tumors. The high-grade non-chemotherapy cohort’s 5-year LC, DC, PFS, and OS are 77.3%, 54.4%, 38.6%, and 62.6%, respectively. The high-grade chemotherapy cohort’s 5-year LC, DC, PFS, and OS are 73%, 68%, 45%, and 70%, respectively (Fig. 3). This is more pronounced when evaluating patients treated with MAI/MAID with a local control, distant control, PFS, and OS of 73.3%, 74.2%, 50.8%, and 76.2%, respectively (Fig. 4). Although a DC benefit was observed in the high-grade chemotherapy arm (p = 0.04), this did not translate into a statistically significant PFS benefit (p = 0.064).

#### Independent predictors of outcome in high-grade subset

On multivariate analysis of the high-grade cohort, chemotherapy independently predicted for a DC benefit (HR 0.475 95% CI 0.236–.954, p = 0.037); however there was no statistical benefit noted on PFS (HR 0.629 95% CI 0.361–1.095, p = 0.101).

In the high-grade cohort, multivariate analysis showed that specifically MAI/MAID chemotherapy independently associated with improvements in DC (HR 0.333 95% CI 0.145–0.767, p = 0.01) and PFS (HR 0.52 95% CI 0.28–0.99, p = 0.047). There was no statistical benefit in OS with chemotherapy (HR 0.564 95% CI 0.248–1.28, p = 0.171) in this subgroup.

## Discussion

Our institutional experience showed patients with high-grade localized STS > 5 cm treated with MAI/MAID chemotherapy had a distant control benefit on univariate and multivariate analysis. However, chemotherapy for all intermediate to high-grade lesions > 5 cm in size was not significantly beneficial in improving distant control in STS. Though the role of chemotherapy in STS has been controversial, our study emphasized grade as an important factor to consider when assessing patients with localized STS > 5 cm for chemotherapy. This finding is consistent with previously identified high risk features commonly associated with distant spread (e.g., high-grade, > 5 cm, increased depth, and location) [13]. This review also emphasizes the importance of good performance status (ECOG 0–1) when making the decision to administer chemotherapy. This factor remained statistically significant in all analyses performed, and overall chemotherapy was well tolerated with only 36% experiencing grade 3 adverse events.

Due to the rarity of STS, studies have typically evaluated the role of chemotherapy in a heterogeneous group, yielding higher power but lacking the ability to identify cohorts that would most benefit from chemotherapy [10, 12]. While the decision to administer chemotherapy can be influenced by patient age, there can be discrepancies when comparing chemotherapy vs. non-chemotherapy cohorts due to age distribution rendering it difficult to accurately calculate an OS benefit. In contrast, evaluating a difference in DC allows us to examine the efficacy of systemic therapy, and is a known predictor to survival detriment [14].

In our study, chemotherapy has an independent and statistically significant DC benefit in patients with high-grade STS of the extremity and trunk. The benefit of chemotherapy was no longer significant when lower risk patients (e.g., intermediate grade sarcomas) were included, but this is likely because our study is underpowered to detect the smaller benefit seen in these patients. When appropriately powered, meta-analyses have shown an OS benefit with the use of perioperative chemotherapy [10, 12]. While a study from the European Organization for Research and Treatment of Cancer (EORTC) did not show an OS benefit with the addition of chemotherapy, this may be attributed to the inclusion of patients with low to intermediate grade sarcoma (55%), which is consistent with our study that the benefit of chemotherapy in the intermediate grade may be less pronounced [11].

Since there is some debate regarding appropriate tumor size cut off for the categorization of high-risk patients (> 5 cm vs. > 8 cm), we evaluated the high-grade cohort for larger tumor size (> 8 cm). Evaluating the smaller cohort of high-grade (n = 210) and size > 8 cm (n = 179) further decreased our power, but chemotherapy independently predicted for a relative 25% distant control and 17% PFS benefit at 5 years. Despite prior evidence of improved chemotherapy responses in patients with pathologic tumor size > 8 cm, our current study did not find a statistically significant benefit with the use of chemotherapy in this cohort (n = 179, p = 0.193) [15, 16].

The first meta-analysis by the Sarcoma Meta-Analysis Collaboration published in 1997 pooled together and reviewed 14 trials (n = 1568) of doxorubicin-based adjuvant chemotherapy, which demonstrated a significant improvement in distant recurrence and progression free survival, with a trend towards improved overall survival. Although this study was adequately powered and illustrated a 5–10% overall survival benefit with doxorubicin-based chemotherapy, it did not clarify which patients would benefit the most from chemotherapy [10, 17]. In 2008, four more studies were added to the Sarcoma Meta-Analysis Collaboration. These studies used Adriamycin with Ifosfamide as the preferred treatment and results showed marginal efficacy of chemotherapy with decreased distant recurrence and improved overall survival [12]. Our study showed that MAI based chemotherapy has a stronger association with a distant control benefit, which translated to a progression free survival benefit and a trend towards a survival benefit at 5 years. Similarly, recent studies evaluating the role of chemotherapy in the treatment of STS have resulted in marginal improvement in disease control and overall survival, with difficulty reaching statistical significance attributed to limitations in sample size, various histologies, and heterogeneous treatment regimens [18–20].

## Limitations

Due to the nature of retrospective cohort studies, there is an inherent bias in this patient population, especially for the choice to deliver chemotherapy. Through multidisciplinary tumor board discussions and recently established treatment pathways, there is an understandable bias for patients to be selected that we believe may benefit more from the addition of chemotherapy to their treatment plan. In addition, the rarity of this disease makes statistical power difficult, especially when evaluating cohorts with lower distant recurrence risk or when evaluating sub-cohorts to identify candidates ideal for this treatment. This study is likely underpowered to show a significant association between chemotherapy use and overall survival, and this may be due to cohort size and early patient censorship. In this cohort, competing risks in survival (e.g., PFS and OS) may be present since we were unable to identify their cause of death.

As with all retrospective studies, subgroup analysis should be interpreted with caution, as analyzing them may raise the type I error. A large prospective multi-institutional study would be required to adequately answer this question, but this can become difficult in this scarce and diverse population.

## Conclusion

To date, the role of chemotherapy in localized STS of the extremity and trunk remains controversial. Expert opinion and current literature review suggests that chemotherapy in this population should be case-based with a preference towards chemotherapy in those that are high-risk. If a patient is deemed high-risk for recurrence, then perioperative chemotherapy is recommended as it has been shown to have a meaningful benefit in outcomes [21]. Although there are many publications to suggest a risk stratification score, standard guidelines have not yet been established [22, 23].

Based on this current study, patients with the highest risk of distant recurrence (i.e., high-grade and > 5 cm) [24] should be strongly considered for perioperative chemotherapy. Specifically, MAI/MAID chemotherapy regimen should be used for appropriate candidates. Although this study was limited in size, the use of chemotherapy showed a significant decrease in distant recurrence for high grade STS of the extremity/trunk > 5 cm, which may translate into an improved overall survival. This should be confirmed with future prospective studies including longer follow-up and more patients.

## Data Availability

The datasets used and/or analysed during the current study are available from the corresponding author on reasonable request.

## Abbreviations

STS: soft tissue sarcoma
LC: local control
DC: distant control
PFS: progression free survival
OS: overall survival
MAI: Mesna, Adriamycin, Ifosfamide
MCC: Moffitt Cancer Center
